# Thrombosis and Coagulopathy in COVID-19: Current Understanding and Implications for Antithrombotic Treatment in Patients Treated With Percutaneous Coronary Intervention

**DOI:** 10.3389/fcvm.2020.599334

**Published:** 2021-01-18

**Authors:** Hangkuan Liu, Zhijia Wang, Haonan Sun, Tianming Teng, Yongle Li, Xin Zhou, Qing Yang

**Affiliations:** ^1^Graduate School of Tianjin Medical University, Tianjin, China; ^2^Department of Cardiology, Tianjin Medical University General Hospital, Tianjin, China

**Keywords:** COVID-19, thrombosis, coagulopathy, antithrombotic treatment, percutaneous coronary intervention

## Abstract

Coronavirus disease 2019 (COVID-19), a respiratory syndrome, is a global pandemic. Therefore, there is an urgent need to explore mechanisms implicated in the pathogenesis of the disease. Clinical and autopsy studies show a complex chain of events preceding COVID-19-related death. The disease is characterized by endothelial dysfunction, platelet activation, thrombosis, coagulopathy, and multiple organ failure. Globally, millions of patients with coronary heart disease undergo percutaneous coronary intervention (PCI) each year. These patients undergo high-intensity antithrombotic therapy during hospitalization and dual antiplatelet therapy (DAPT) for at least 6 months post PCI. COVID-19 is characterized by changes in platelet counts. Treatment of ischemic events that occur during stent implantation is associated with bleeding complications in patients following PCI complicated by COVID-19. This review summarizes recent progress in activation status and levels of COVID-19-related platelet changes. These findings will provide information on the effectiveness of antithrombotic therapy for the management of platelet changes in COVID-19 patients.

## Introduction

Coronavirus disease 2019 (COVID-19), a severe acute respiratory syndrome coronavirus 2 (SARS-CoV-2) infection, is a worldwide pandemic. In November 2020, the World Health Organization reported over 50 million confirmed cases of COVID-19 and 1.3 million deaths globally (1). COVID-19 is associated with pneumonia and a wide range of effects on the cardiovascular system, thus, it is a health and economic burden worldwide (2–5).

SARS-CoV-2 enters host cells through coupling of viral spike protein and angiotensin-converting enzyme 2 (ACE-2) on the surface of host cells (6, 7), in a similar way as observed during SARS-CoV infection (8). Previous autopsy evaluations of SARS-infected patients (9) and recent clinical trials on COVID-19 patients (10, 11) show that diffuse alveolar injury and development of acute respiratory distress syndrome (ARDS) are the main pulmonary pathological manifestations. Cardiovascular effects, especially venous thromboembolic disease (12, 13) and ischemic complications in arterial system, such as ischemic stroke (14), have been reported in COVID-19 patients.

A recent study reports significant changes in platelet gene expression and function in COVID-19 patients. These changes result in platelet activation and aggregation, which are potential novel mechanisms for management of COVID-19-associated thrombosis and coagulopathy (15). Notably, severe COVID-19 cases present with thrombocytopenia (16), which is associated with platelet depletion and a high risk of bleeding. Approximately 5 million percutaneous coronary interventions (PCIs) are performed each year worldwide (17). Therefore, COVID-19 patients requiring antithrombotic therapy have a high risk of thrombotic events and bleeding complications (16). Hence, in this review, we explored recent studies reporting relationships between changes in platelet function and coagulopathy in COVID-19 patients. The findings of this study will provide a mechanistic basis for designing new treatment approaches for thrombosis and coagulopathy in COVID-19 patients. Further, this study provides information for the development of personalized antithrombotic therapy regimen for COVID-19 patients treated with PCI.

## Clinical Characteristics of COVID-19 Patients

A previous clinical trial reports that the prevalence of hypertension, diabetes, and coronary heart disease among COVID-19 patients in the first 2 months of the outbreak was 15, 7.4, and 2.5%, respectively (18). Prevalence of hypertension, diabetes, and coronary heart disease significantly increased to 35.8, 26.9, and 9.0%, respectively, for patients who were admitted in intensive care units receiving mechanical ventilation or patients who succumbed to the disease (18). In a study carried out at Mount Sinai Hospital, comorbidity with hypertension (62.7%), diabetes mellitus (40.3%), coronary artery disease (31.3%), chronic kidney disease (26.7%), and asthma (17.9%) was higher in patients who succumbed to COVID-19 compared with that of survivors (19). Notably, thrombocytopenia (defined as a platelet count of <150,000/μl) was observed in 36.2% patients on admission, mainly in patients with severe cases (18). Moreover, prolonged prothrombin time and elevated D-dimer level, which indicated coagulopathy associated with COVID-19, were mainly reported in severe cases (3, 18, 20).

SARS-CoV-2 is a new coronavirus strain that belongs to the same class with SARS reported in 2003 (21). Clinical studies on SARS patients reported an increase in activated partial thromboplastin time (42.8%), thrombocytopenia (44.8%), and elevated D-dimer (45.0%) (22). In addition, a previous study reports thrombocytopenia in SARS patients (55%), increase in activated partial thromboplastin time (63%) and disseminated intravascular coagulation (DIC, 2.5%) (23). Clinical manifestations observed in SARS-CoV- and SARS-CoV-2-infected patients indicate a high risk of DIC. Therefore, World Health Organization interim guidance statement recommends prophylactic administration of low-molecular-weight heparin daily or subcutaneous administration of unfractionated heparin 2 times in a day (24). In addition, American College of Cardiology recommends that patients should receive all scheduled doses of venous thromboembolism prophylaxis (25). Administration of low-molecular-weight heparin daily is preferred over unfractionated heparin, as it reduces personal protective equipment use and exposure of health care workers (25).

## Brief Summary of Viral Pneumonia Pathology

Viral pneumonia accounts for one third of adult community-acquired pneumonia. Most viral pneumonia cases are caused by influenza, rhinovirus, and coronavirus infections (26). Viral pneumonia is characterized by histopathological changes including interstitial pneumonitis with lymphocytic infiltrations. Other manifestations such as necrotizing bronchiolitis, diffuse alveolar injury with alveolar hemorrhage, alveolar septal edema, and hyaline-membrane formation may be present depending on conditions associated with co-infection and underlying disease (26).

### Lung Pathology of Severe Acute Respiratory Syndrome

During 2002 and 2003, the SARS-CoV caused severe respiratory infection in more than 8,000 people and led to 774 deaths, with a mortality rate of 9.6% (27). The typical pathological change in SARS-infected lungs was diffuse hemorrhage on the lung surface and serous, fibrinous, and hemorrhagic inflammation in most pulmonary alveoli (9). In addition to diffuse alveolar hemorrhage, other commonly observed findings were the presence of intra-artery fibrin thrombi (5/8) and intra-alveolar hemorrhage (6/8) (28).

### Lung Pathology and Multiple Organ Failure in Coronavirus Disease 2019

The early pulmonary pathological changes in SARS-CoV2-infected lungs included edema, proteinaceous exudate, and focal reactive hyperplasia of pneumocytes with patchy inflammatory cellular infiltration, whereas hyaline membranes were not prominent (29). The key features of lung pathology from severe COVID-19 patients were bilateral diffuse alveolar injury with cellular fibromyxoid exudates, as well as hyaline membrane formation (11). Other pathological findings included the presence of inflammatory lesions (gray-white lesions), dark red bleeding lesions, and sticky secretions in the lung tissue (10) and severe alveolar edema and hemorrhagic necrosis in both lungs, along with extensive pulmonary interstitial fibrosis and partial hyaline degeneration (30). These findings provided clear evidence for diffuse alveolar injury in severe COVID-19 cases.

There have been accumulating pathologic findings of COVID-19 outside China (12, 31–34). Diffuse alveolar injury, endothelial injury, thromboembolism, and viral particles within renal cells were reported in various COVID-19 cases (12). A case series from Washington State showed that coronavirus-like particles were detected in the respiratory system, kidney, and gastrointestinal tract (32). Additionally, in one patient complicated by myocarditis, the viral RNA could be detected in the heart as well (32). More recent findings suggest that COVID-19 may be a complex infection associated with extensive vascular endotheliitis (33, 34), manifesting an imbalance between the coagulation and immune functions in the body, which would pose the infected individual at risk for developing multiple organ failure (MOF).

It should be noted that the above pathological findings are mainly the direct and indirect consequences of lung tissue destruction induced by intracellular viral proliferation. In the rapid progressive and life-threatening form of viral pneumonia, the underlying pathological process is often diffuse alveolar injury, coagulopathy, and MOF (26). Collectively, these findings revealed a mechanistic link between virus infection, proliferation, and diffuse alveolar injury (35): the pathologic change evolves from alveolar capillary dysfunction and platelet activation, followed by intravascular fibrin and micro-thrombus formation; if left uncontrolled, these alterations would trigger systemic dissemination and secondary fibrinolysis and result in platelet and coagulation factor depletion and consequently lead to DIC, even MOF.

## New Mechanisms Underlying Thrombosis and Coagulopathy in COVID-19

**Figure 1:**
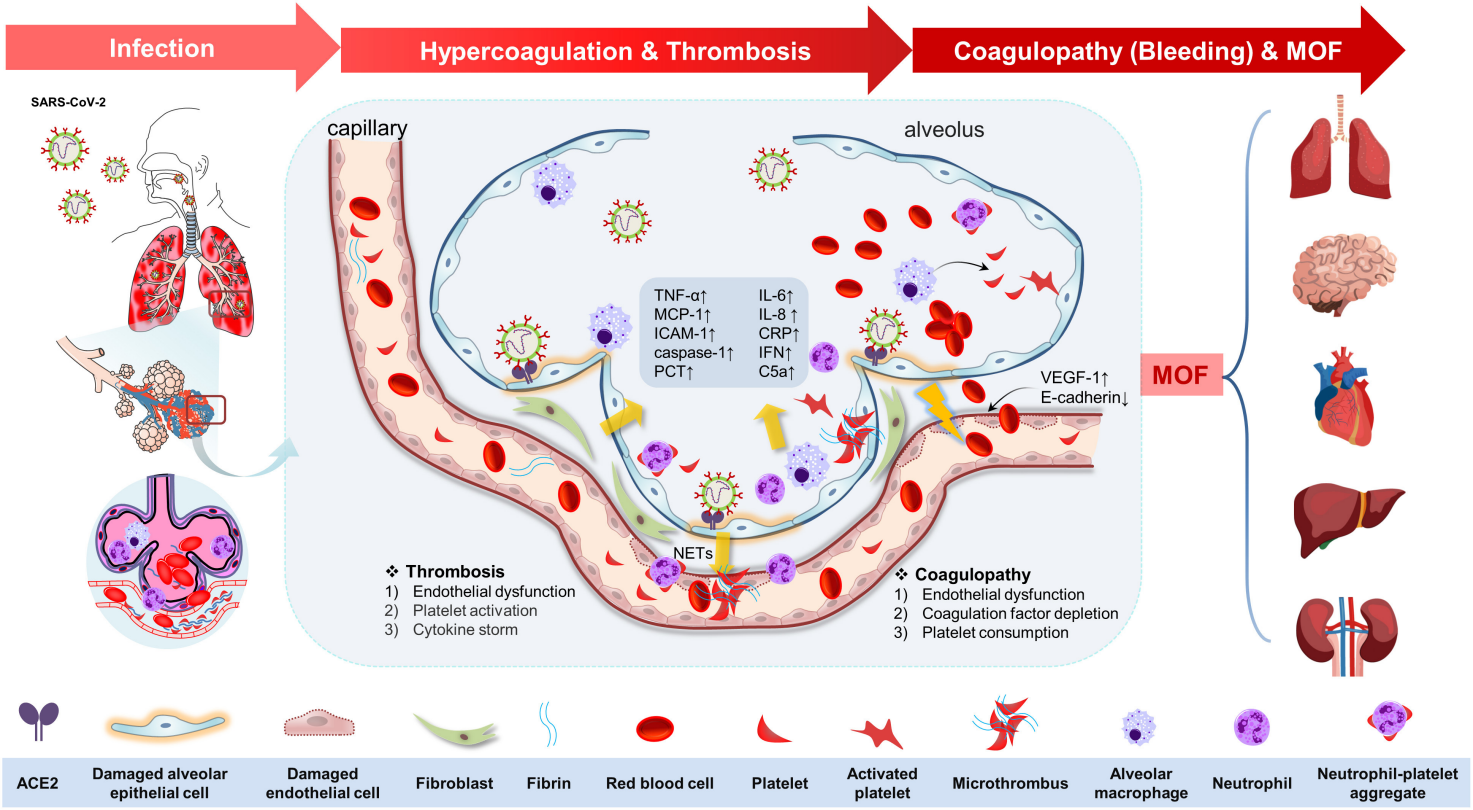
The potential pathophysiological evolutions underlying severe acute respiratory syndrome coronavirus 2 (SARS-CoV-2) infection, linking pulmonary inflammation, multiple organ failure, and thrombosis and coagulopathy. The potential pathophysiological evolutions underling SARS-CoV-2 infection was summarized as the following three stages: infection, hypercoagulation, and thrombosis, coagulopathy (bleeding tendency), and multiple organ failure. First, the SARS-CoV-2 could transmit through the respiratory tract in the infected host by angiotensin-converting enzyme 2 (ACE-2) in the epithelial cells in the trachea or lung tissues. The viral proliferation and dissemination within the lung tissue lead to *in situ* endothelial cell injury and platelet and immune system activation. Second, endothelial dysfunction [upregulation of vascular endothelial growth factor receptor 1 (VEGF-1) and downregulation of E-cadherin] elicits the inflammation response and platelet activation to seal the damaged endothelium. Platelet activation was mediated by mitogen-activated protein kinase (MAPK) pathway activation and thromboxane generation, which further induces neutrophil extracellular trap (NET) formation, contributing to the microthrombus with fibrin. Additionally, immune defense and cytokine storm also participate in this process by means of immunothrombosis. This process is characterized by upregulation of tumor necrosis factor-α (TNF-α), monocyte chemotactic protein 1 (MCP-1), intercellular cell adhesion molecule-1 (ICAM-1), caspase-1, interleukin-6 (IL-6), IL-8, C-reactive protein (CRP), interferon (IFN), C5a, procalcitonin (PCT), etc. Antiplatelet therapy is of therapeutic potential at this stage. Third, coagulopathy (bleeding tendency) was associated with endothelial dysfunction, coagulation factor depletion (including fibrinogen and others), and platelet consumption. Finally, all of these changes in COVID-19 drive the progress of multiple organ failure (MOF), including brain, heart, lung, liver, and kidney.

Replication and dissemination of SARS-CoV-2 in systemic circulation lead to extrapulmonary manifestations, which play key roles in disease progression (2, 34, 36). A previous German prospective cohort study reports a high incidence of deep venous thrombosis (58%) and diffuse alveolar injury (67%) (12). These manifestations are associated with enhanced inflammatory state and hypercoagulable state, resulting in higher rates of venous and arterial thrombosis (12, 37). Moreover, increased severe bleeding rates are reported in critically ill patients following preventive or therapeutic anticoagulant and antiplatelet therapy (37). Subsequent sections of this review will summarize mechanisms involved in the pathogenesis of thrombosis and coagulopathy in COVID-19 patients as a complex chain of pathophysiological events preceding COVID-19-related death (summarized in Figure 1).

### Endothelial Dysfunction

Endothelial dysfunction induces inflammation and vascular remodeling (38), which are associated with severe COVID-19. Endotheliopathy or endothelial dysfunction, including endothelial activation, endotheliitis, and thrombotic events, is an indicator of coagulopathy in COVID-19 patients (33, 34, 39).

SARS-CoV-2 enters host cells by binding to ACE-2 on pulmonary epithelial cells resulting in lung damage (6, 7). Moreover, vascular endothelial cells of multiple organs, including kidney, heart, and small bowel are infected by SARS-CoV-2 directly. Infection induces apoptosis and pyroptosis, which result in diffuse endothelial inflammation (40). Upregulation of vascular endothelial growth factor and downregulation of E-cadherin expression enhance the permeability of endothelial cells in COVID-19 patients (41). In addition, biopsy of lung tissues of COVID-19 patients shows upregulation of interleukin-6 (IL-6), tumor necrosis factor-α, intercellular adhesion molecule-1, and caspase-1 expression (39). Further, quantitative analysis showed a significant increase in expression levels of von Willebrand factor antigen and soluble P-selectin, which are markers of endothelial cell and platelet activation, in COVID-19 patients admitted to intensive care units (34). Notably, increased expression levels of von Willebrand factor antigen and soluble P-selectin are correlated with mortality (34). Increases in expression levels of these markers imply that endotheliopathy is implicated in the pathogenesis of COVID-19.

### Platelet Activation and Depletion

A previous study reports that lungs have a high hematopoietic potential, thus they contribute to terminal platelet production (nearly 50%) (42). Therefore, the platelet-related response in COVID-19 patients may be more rapid and severe during the initial stage of pulmonary infection. In addition to thrombosis and hemostasis, previous studies report a putative role of platelets in host defense against infections (43–45). A previous study using a mouse model reports that platelets migrate to the microvasculature (46). Migratory phenotype contributes to mechano-scavenging and bundling of bacteria and boosts innate immunity in a mouse model of severe bacteremia (46). Moreover, human and murine platelets are induced by a range of antimicrobial compounds, especially platelet microbicidal proteins to exert direct microbicidal activity (43). These findings make it challenging to interpret the impact of platelet activation and depletion in COVID-19 patients. Reports from observational studies during the early days of the outbreak in China and other countries show a significant change of platelet response (from excessive activation to depletion) during the progression of severe COVID-19 cases (15, 47).

At the early phase of infection, SARS-CoV-2 invades the lung tissue of the host, which may activate platelets through changes in gene expression (15, 42). Platelets detect invading pathogens through a broad array of receptors and elicit interaction with immune cells (neutrophils, monocytes, and lymphocytes) (15). Activated platelets then exhibit an augmented aggregation capacity by upregulating membrane P-selectin level, which enhances interactions and aggregation with neutrophils, monocytes, and T cells, through mitogen-activated protein kinase (MAPK) pathway activation and thromboxane generation (15).

Lungs and bone marrow are the main sources of compensatory production of platelet consumption in a variety of thromboembolic disorders (42). During infection, neutrophil extracellular trap formation, platelet aggregation, and microthrombus formation reduce tissue perfusion and aggravate inflammation and endothelial injury by activating leukocyte signaling (48). A previous study showed that a high bleeding rate in critically ill COVID-19 patients (7.6 vs. 3.1%) was positively correlated with peak D-dimer levels and negatively correlated with platelet counts (49). Replenishment of circulating platelets is a fine-tuned process determined by the dynamic balance between platelet consumption and production. In severe pulmonary inflammatory response, such as SARS and COVID-19 cases, virus proliferation and dissemination within the lung tissue may directly contribute to *in situ* activation of lung megakaryocyte-derived platelets or have a direct impact on lung megakaryocytes. This may lead to changes in gene expression profile of platelets as observed in COVID-19 patients (15). If this response is not resolved within the lung tissue, platelet activation and ensuring microthrombus formation in lung vasculature would further aggravate pulmonary inflammation. Systemic endothelitis caused by dissemination of SARS-CoV-2 then elicits a second wave of platelet activation in extrapulmonary organs. The second wave leads to a more severe form of platelet activation resulting in consumptive thrombocytopenia (18). Therefore, there is a critical transition in which the beneficial effect of antiplatelet therapy at an early stage of COVID-19 can be attenuated or may have severe effects by aggravating bleeding when clinically significant thrombocytopenia develops.

### Immune Defense and Cytokine Storms

Activation of the immune system, which includes production of cytokines, immune complements, and various immune cells, plays an important role in fighting SARS-CoV-2 infection (41). Immune response is always a double-edged sword. Under inflammatory conditions in COVID-19 patients, immunothrombosis, which contains invading pathogens driven by platelets, neutrophils, and the coagulation cascade, is a central pathogenic factor linking respiratory failure and systemic hypercoagulation (50). In addition, immunothrombosis leads to vessel occlusion and tissue hypoxia, which may enhance the inflammatory response. COVID-19 patients with loss-of-function variants of Toll-like receptor 7, which mediates type I interferon and interferon-γ production, show poor prognosis and subtle subsegmental pulmonary embolisms (51). These findings imply that the immune system is closely associated with thrombosis during SARS-CoV-2 infection.

Recent studies report that the serum of COVID-19 patients showed elevated cytokine levels (C-reactive protein, IL-6, IL-8, and monocyte chemotactic protein-1) (41), high complement levels (C5a) (52), and reduced lymphocyte counts (41). The negative correlation between high levels of IL-6 or IL-8 and low lymphocyte counts indicates underlying mechanisms that link these characteristics in severe disease, including immunothrombosis. This finding is consistent with reports that treatment of COVID-19 patients with tocilizumab, which blocks IL-6-mediated signaling, restored circulating levels of lymphocytes to levels close to normal ranges (53). Enhanced host immune response plays a pivotal role in inducing MOF. Therefore, the efficacy of dexamethasone, a nonspecific immunosuppressant, was evaluated in a large randomized clinical trial and a meta-analysis in patients hospitalized with COVID-19 (54, 55). The results showed approximately 30% reduction in mortality for patients under respiratory support. Therefore, these findings imply that excessively activated host immune response aggravates COVID-19-associated MOF.

## Antithrombotic Therapy in COVID-19 Patients Treated with Percutaneous Coronary Intervention

During the COVID-19 global pandemic lockdown period, the number of patients presenting with acute coronary syndrome (ACS) and emergency coronary procedures reduced significantly in Europe (56–58), the USA (59, 60), and Asia (61). However, ACS patients were the main target population among patients with coronary heart disease in cardiology departments during the COVID-19 epidemic compared with patients with chronic coronary syndrome (62). At the beginning of the COVID-19 outbreak, the number of admitted ACS patients in most world regions significantly reduced. Clinical management of ACS during this period was characterized by a decrease in hospitalization rate [−48.4% in Italy (57)], a decrease in PCI rate [−24% in China (61), −43% in Hubei (61), −32% in Italy (63)], and an increase in thrombolytic rate [+66% in China (61), +378% in Hubei (61)]. A recent study in UK showed that a reduction in ACS hospitalization by 40% from the initial days of the COVID-19 outbreak was gradually decreasing to a 16% reduction in May 2020 (64). The number of PCI procedures decreased in both ST-segment elevation myocardial infarction (STEMI) and non-STEMI patients (−21 and −37%, respectively) (64). Furthermore, STEMI patients with COVID-19 showed a higher thrombus load, with 17.9% of these patients presenting with multiple thrombus formation (65). In addition to ensuring timely and effective revascularization of ACS patients (especially STEMI patients), the control of COVID-19 infection in ACS patients is important. Different countries have different views on treatment approaches of ACS patients coinfected with COVID-19 (66–73). Therefore, there is a need to explore appropriate treatment measures for ACS patients during the COVID-19 epidemic.

### Coronavirus Disease 2019-Related Delay: The Dilemma for Pre-hospital Management of Acute Coronary Syndrome

Early diagnosis and timely management are critical in reducing morbidity and mortality related to ACS. Ischemic time duration is a major determinant of infarct size in patients with STEMI. Current delays in COVID-19 testing, termed as “COVID-19-related delay,” may contribute to total ischemia time (74). Tam et al. (75) reported that median pre-hospital delay increased from 82.5 to 318 min and door to device time increased from 84.5 to 110 min.

### Primary Percutaneous Coronary Intervention or Thrombolysis: The Choice of Optimal In-hospital Treatment of Acute Coronary Syndrome

Recent studies showed that patients with STEMI presenting with concurrent COVID-19 present with unique findings during coronary angiography (65, 76). A study carried out in Italy reports that 11 patients (39.3%) out of 28 COVID-19 patients admitted for STEMI showed no obstructive coronary artery disease (76). Another single-center study from UK comprising 115 consecutive STEMI patients with confirmed concurrent COVID-19 reported significantly higher rates of multivessel thrombosis, stent thrombosis, and glycoprotein IIb/IIIa inhibitor use (65). Notably, these findings were based on small observational studies. However, angiographic manifestations require a dedicated diagnostic approach and a modified antithrombotic regimen for this special population.

While primary PCI remains the treatment of choice for STEMI, the balance between exposure risk of medical staff and benefit of patient from thrombolysis should be considered in certain circumstances. Strategic Reperfusion Early After Myocardial Infarction study demonstrated that even a single hour of delay may affect the effectiveness of primary PCI compared with thrombolysis (77).

In China and Iran, thrombolytic therapy is recommended over primary PCI for STEMI management if COVID-19 was confirmed or could not be excluded within a short time. On the other hand, SARS-CoV-2 infection is excluded first for non-STEMI and unstable angina pectoris approaches (66–69). Conversely, organizations from the United States (72), Europe (73), Australia, and New Zealand (70) recommend the use of existing primary PCI protocols for STEMI patients except for confirmed COVID-19 patients and persons under investigation or cases in which primary PCI could not be performed within required time frames. Moreover, previous studies recommend that coronary angiography should be performed prior to discharge after the patient has stabilized from COVID-19 (78).

### Periprocedural Anticoagulant Therapy: Intensified and Prolonged

A high risk of thrombotic complications in patients with COVID-19 complicates the dosage of anticoagulation in hospitalized patients with COVID-19 (25). Anticoagulation is recommended for patients with thrombotic complications in addition to antiplatelet therapy during primary PCI (79, 80). In addition, routine use of unfractionated heparin (I, C) and enoxaparin intravenous (IIa, A) should be considered (79). In patients with heparin-induced thrombocytopenia, bivalirudin is recommended as an anticoagulant agent during primary PCI (I, C) (79). The optimal dosage of anticoagulants (conservative or radical) in COVID-19 patients should be personalized based on inflammatory state and a hypercoagulable state of the patients.

The 2018 European Society of Cardiology guideline does not give guidelines on routine post-procedural anticoagulant therapy after primary PCI (79). STEMI patients should receive at least 48 h of anticoagulation therapy after intravenous thrombolysis (80). Introduction of post-procedural anticoagulation and prolongation of anticoagulation therapy is required to counterbalance the COVID-19-related systemic hypercoagulability after primary PCI and intravenous thrombolysis for COVID-19 patients. Notably, these therapy approaches may increase the risk of heparin-induced thrombocytopenia (81).

### Dual Antiplatelet Therapy: The Choice of Optimal P2Y_12_ Inhibitor

In a previous prospective study, our group summarized reports on thrombotic and bleeding incidence from early findings of COVID-19 outbreak and reported on the pros and cons of antithrombotic treatment for patients following PCI (16). The findings from this review add more information on the use of antithrombotic treatment in COVID-19 patients. Notably, the time of initiating an antithrombotic regimen should be considered. In the early phase of COVID-19, platelet inhibition by dual antiplatelet therapy (DAPT) may suppress the hyperactivation state of platelets probably through inhibition of *in situ* platelet activation in lung vasculature (15, 42). Antiplatelet agents used at this stage affect intravascular fibrin and thrombus formation, thereby preventing secondary fibrinolysis and coagulation factor depletion. Notably, observational studies report that pre-hospitalization aspirin use is associated with lower mortality in patients with community-acquired pneumonia (100 mg) (82) and ARDS (75–300 mg) (83). On the contrary, findings from a randomized clinical trial show that aspirin administration after admission (325 mg loading followed by 81 mg daily for 7 days) does not prevent the development of ARDS (84). In addition, the choice of antiplatelet agents with different intensity modulates the effectiveness of antithrombotic treatment. An observational study showed that pre-hospital exposure to clopidogrel is associated with an increased risk for community-acquired pneumonia (85). Most patients treated with clopidogrel would receive aspirin. Therefore, it is difficult to draw a conclusion that P2Y_12_ inhibitor is harmful in terms of pneumonia prevention. However, higher intensity of platelet inhibition may lead to the suppression of antimicrobial effect of platelets. Furthermore, discontinuation of aspirin 1 to 3 months after PCI with continued P2Y_12_ inhibitor monotherapy significantly reduces the risk of major bleeding by 40~50%, with no increased risk of major adverse cardiovascular events, compared with traditional DAPT (86). Therefore, continued P2Y_12_ inhibitor monotherapy may be relatively safe after PCI in COVID-19 patients with a higher risk of bleeding. Ticagrelor, a unique P2Y_12_ inhibitor, has an additional target of inhibition, the equilibrative nucleoside transporter 1; therefore, it results in higher antiplatelet effects and antibacterial activity (87). Moreover, a clinical benefit of ticagrelor in the management of pneumonia by preventing sepsis complications and reducing lung injury was reported in the recent XANTHIPPE (Targeting Platelet-Leukocyte Aggregates in Pneumonia With Ticagrelor) trial (88) and PLATO study (89). Furthermore, clinicians should carefully evaluate platelet counts and levels of other hematological parameters when describing antiplatelet agents. Both primary (idiopathic thrombocytopenic purpura) and secondary thrombocytopenia (enhanced consumption) are associated with an increased risk of infection (including pneumonia) (90), poor outcomes associated with pneumonia (91, 92), and increased mortality for ARDS (93). Individuals who are thrombocytopenic lose the ability to deposit fibrinogen and fail to seal damaged pulmonary vasculature (94). Therefore, platelets are potential therapeutic targets to help predict the onset of ARDS. Currently, there are no available studies on prolongation and intensified antiplatelet therapy in reducing COVID-19-related thrombosis and MOF; therefore, antiplatelet therapy, especially ticagrelor, following PCI should be maintained. However, a study carried out on East Asian populations showed a significantly higher incidence of clinical bleeding in the ticagrelor group compared with that in the clopidogrel group (11.7 vs. 5.3%; hazard ratio, 2.26; 95% confidence interval, 1.34–3.79; *P* = 0.002) (95). A recent study from the SWEDEHEART Registry reports that ticagrelor use among elderly ACS patients is associated with a higher risk of bleeding (hazard ratio, 1.48; 95% confidence interval, 1.25–1.76) and death (hazard ratio, 1.17; 95% confidence interval, 1.03–1.32) compared with the use of clopidogrel (96). Therefore, ticagrelor should not be prescribed to the elderly and East Asian populations. Moreover, the balance between platelet consumption and production, host immune response, and the fact that the clinical benefit of DAPT in the context of COVID-19 is dependent on the severity of the disease should be considered during treatment.

### Prognosis of Acute Coronary Syndrome Patients Presenting With Concurrent Coronavirus Disease 2019

Currently, the long-term impact of COVID-19-related endothelial activation, hypercoagulability, microvascular thrombosis, and myocardial injury is not well-known (97). Previous studies report that multiple imaging techniques can accurately assess cardiovascular conditions in COVID-19 patients (98–101). Cardiac nuclear magnetic resonance in patients recovering from COVID-19 infection shows myocardial involvement (mainly myocarditis, including myocardial edema, fibrosis, and impaired right ventricular function) (99–101). Interestingly, a 12-year follow-up survey of 25 patients who recovered from SARS-CoV infection showed that 68% of these patients had hyperlipidemia, 44% had cardiovascular system abnormalities, and 60% had glucose metabolism disorders (102). SARS-CoV-2 and SARS-CoV mechanism of infection and systemic involvements are similar; therefore, long-term prognosis of COVID-19 patients should be explored.

Zhang et al. (103) reported that in-hospital use of statins among 13,981 cases of COVID-19 was significantly associated with a lower risk of death (5.2 vs. 9.4%, adjusted hazard ratio of 0.58) and less inflammatory response during the entire hospitalization period compared with non-statin use. This finding implies that statin plays a protective role in the acute management of COVID-19 by protecting vascular endothelium and regulating immunity (104).

## Conclusion

Findings from clinical observations and autopsy studies show a complex chain of events preceding COVID-19-related death. The adverse event chain starts with viral infection and proliferation, followed by endothelial dysfunction induced by local and systemic viral dissemination. Further, platelet activation, thrombosis, and platelet and coagulation factor depletion occur leading to MOF and life-threatening bleeding. Patients treated with PCI and patients on antithrombotic treatment should undergo post-procedural anticoagulation and prolonged anticoagulation following intravenous thrombolysis and standard DAPT treatment to reduce the risk of thrombotic complications during early-to-mid stages of COVID-19 progression. Pros and cons of these antithrombotic treatment regimens should be evaluated in an individualized manner in cases of clinical thrombocytopenia (induced either by platelet consumption or by heparin) and/or bleeding complications.

## Author Contributions

HL and ZW performed the manuscript writing and illustration drawing. HS performed part of literature collecting. TT and YL provided critical scientific input and discussions. XZ and QY conceived, designed, and supervised all studies and the drafting and editing of the manuscript. All authors contributed to the article and approved the submitted version.

## Conflict of Interest

The authors declare that the research was conducted in the absence of any commercial or financial relationships that could be construed as a potential conflict of interest.

## Abbreviations

ACS: acute coronary syndrome
ARDS: acute respiratory distress syndrome
COVID-19: coronavirus disease 2019
DAPT: dual antiplatelet therapy
DIC: disseminated intravascular coagulation
IL-6: interleukin-6
MOF: multiple organ failure
PCI: percutaneous coronary intervention
SARS-CoV-2: severe acute respiratory syndrome coronavirus 2
STEMI: ST-segment elevation myocardial infarction.
